# Comparison of smoking habits, knowledge, attitudes and tobacco control interventions between primary care physicians and nurses

**DOI:** 10.1186/s12971-015-0062-7

**Published:** 2015-11-12

**Authors:** Cemil Isik Sonmez, Leyla Yilmaz Aydin, Yasemin Turker, Davut Baltaci, Suber Dikici, Yunus Cem Sariguzel, Fatih Alasan, Mehmet Harun Deler, Mehmet Serkan Karacam, Mustafa Demir

**Affiliations:** Department of Family Medicine, Duzce University, School of Medicine, Duzce, Turkey; Department of Chest Diseases, Duzce University, School of Medicine, Duzce, Turkey; Community Health Center, Isparta, Turkey; Department of Neurology, Duzce University, School of Medicine, Duzce, Turkey; Community Health Center, Duzce, Turkey; Cukurca State Hospital, Hakkari, Turkey; Poturge State Hospital, Malatya, Turkey; Tuzla Family Health Center, Istanbul, Turkey

## Abstract

**Background:**

Primary care providers are uniquely positioned to initiate smoking cessation. We aimed to evaluate knowledge levels about the health effects of smoking and attitudes toward smoking and tobacco control activities among primary care providers.

**Methods:**

In the cross-sectional and primary care-based study, self-administered surveys modified from the WHO Global Health Professional Survey 5A steps of smoking cessation practice (Ask, Advise, Assess, Assist and Arrange) were provided to primary care physicians (PCPhs) and nurses (PCNs).

**Results:**

Respondents included 1182 PCPhs and 1063 PCNs. The proportions of current and former smokers were significantly higher among PCPhs than among PCNs (34.4 vs. 30.7 % and 14.0 vs. 10.1 %, respectively; both *P* < 0.001). We observed that 77.2 % of PCPhs and 58.4 % of PCNs always or rarely practiced an “Ask” step about their patients’ smoking status (*P* < 0.001). One-third of PCPhs (33.8 %) stated that they always practiced an “Ask” step, whereas only 27.6 % of PCNs always did so in their practice (*P* < 0.001). A small minority of primary care providers had advised patients to quit smoking, although there was a significant difference in this between PCNs and PCPhs (8.4 vs. 15.6 %; *P* < 0.001). Most PCPhs considered themselves competent in advising about smoking interventions, but only a minority of PCNs did so (75.1 vs. 17.3 %; *P* < 0.001). Among barriers to tobacco intervention measures, lack of time was the item most commonly cited by PCPhs, whereas low patient priority was most commonly cited by PCNs (35.9 and 35.7 %; *P* < 0.001).

**Conclusions:**

Smoking intervention practice by primary care nurses was quite low. Lack of time and low patient priority were identified as barriers by primary care providers. Strategies by which primary care providers could improve tobacco control should be established.

## Introduction

Smoking is a common and leading preventable cause of mortality and morbidity in population worldwide. Intervention against tobacco use is one of the most public problems preferably identified by WHO at primary health care. Health care professionals, primarily primary care providers (PCPs), are expected to contribute to tackle this public problem [1]. The struggle with tobacco requires cooperation and collaboration of policy, health professionals and public incorporation. PCPs, including primary care physicians (PCPhs) and primary care nurses (PCNs), are corner stones in tobacco control, and play a major part in providing smoking intervention [2]. They are in a gateway position, and so are expected to be the most important providers for smoking cessation practice [3]. A systematic review showed that the majority of PCPs doesn’t hold negative beliefs and attitudes towards discussing SCP with their patients. Readiness, competence and confidence of PCPs for SCP increase a patient’s chance to succeed in quitting smoking. Smoking can affect preparedness, engagement and priority of PCPs [4]. However, smoking habits, attitudes and skills of primary care providers determine their smoking cessation practice. The frequency of smoking among PCPs is not rare, and this undermines their roles and practice [5, 6].

It is essential that health care providers consistently identify and document the status of smoking and appropriately practice smoking intervention for every tobacco user encountered in a health care setting, not only the ones already suffering from tobacco-related diseases. It was suggested that there were many reasons for PCPs to fail in smoking intervention, such as lack of knowledge about how to identify smokers quickly and easily, time constraints, incompetence, limited training in tobacco cessation, or lack of reimbursement [7, 8].

Both primary care nurses and physicians are almost equally responsible for tobacco interventions, although they are different health care groups. A few developed countries such as England, Netherland and Denmark have established tobacco control in their primary care settings and have vested both primary care nurses and physicians with authority for smoking cessation practice. Non-smoking by health professionals is a model behavior for their patients as well as for the general public. Ratio of tobacco use among health professionals has decreased in many developed countries in the last 20 years [9]. Beside the successful strategy and policy against tobacco use, its prevalence remains high in Turkey according to The Report 2010 of Health Ministry of Turkey and Global Youth Tobacco Survey. They notified that smoking use among PCPs were about 30 % and 31 % [10, 11]. The purpose of the study was to evaluate and compare the knowledge level about health effects of smoking, their attitudes towards smoking and anti-tobacco control activities between primary care providers in Turkey.

## Methods

### Study design and data collection

The study protocol was described in a previous publication by Baltaci et al. [12]. The target group was primary care providers, including primary care physicians (PCPhs) and primary care nurses (PCNs), working in primary care settings through Turkey. A structured questionnaire modified from the Global Health Professional Survey, originally developed by the World Health Organization (WHO), was used. The survey was given to PCPhs and PCNs in primary care settings and was self-administered. The study surveys were distributed to 1500 PCPhs and 1500 PCNs, mostly as handouts (94.2 %). In total, 1233 surveys from PCPhs (response rate 82.2 %) and 1340 surveys from PCNs (response rate 89.3 %) were returned. Surveys from 12 PCPhs and 17 PCNs with missing data were excluded.

The survey covered smoking habits, basic socio-demographic information, smoking intervention skills, knowledge about smoking’s effects, barriers to smoking cessation practices, attitudes toward smoking, and intention to quit smoking among PCPs. Knowledge level was evaluated with five items regarding the harmful effects of smoking, and attitudes were evaluated with 15 items regarding the physician’s role in tobacco control and anti-smoking activities. The Fagerstrom nicotine dependence test (FNDT) was applied to current smokers. Barriers to tobacco intervention were evaluated with questions asking about four possible barriers: lack of time, low patient priority, low provider priority, and lack of reimbursement. All information was analyzed, and results for PCPh and PCN participants were compared.

### Ethics

Legal permission for the study was provided by the Department of Family Medicine, Institution of Public Health, Ministry of Health, Republic of Turkey. Before completing the survey, all providers were informed about the study by a cover sheet. Participation in the study was voluntary. The study was approved by the Ethics Committee of Medical Faculty, Duzce University, and was in accordance with the ethical standards laid down in the Declaration of Helsinki (1964). The questionnaire was anonymous, and confidentiality of the data was maintained.

### Statistics

Statistical analyses were performed using SPSS software (ver. 20.0 for Windows, SPSS, Inc., Chicago, IL, USA). Continuous variables are expressed as mean ± standard deviation and categorical variables were stated as percentage and frequency. Comparisons of PCPh and PCN results for categorical variables were performed using the *χ*^2^ test or Fisher’s exact test. Continuous variables were tested for a normal distribution using the Kolmogorov–Smirnov test. Comparisons of continuous variables with normal distributions were made using the Student’s *t*-test. Variables that were not normally distributed were log-transformed, and then the Student’s *t*-test was used. A *p*-value <0.05 was considered to indicate statistical significance.

## Results

Respondents included 1182 PCPhs (male: 59.6 %, female: 43.1 %) and 1063 PCNs (male: 8.2 %, female: 91.8 %) who had been working in primary care settings. The mean ages of the PCPhs and PCNs were 38.8 ± 6.8 and 33.6 ± 6.3 years, respectively (*P* < 0.001).

Table 1 provides information about the smoking habits of the primary care providers. The frequency of current smokers among male participants in both groups was higher than that for female participants (*P* = 0.001 and 0.001, respectively). The proportions of current and former smokers were significantly higher among PCPhs than among PCNs (34.4 vs. 30.7 % and 14.0 vs. 10.1 %, respectively; *P* < 0.001). Smoking duration, age at giving up smoking, and number of cigarettes per day among PCPhs differed significantly from those for PCNs (*P* < 0.001, < 0.001, and < 0.001, respectively). Fagerstrom nicotine dependence test (FNDT) scores among current smokers were similar between these groups (3.7 ± 2.7 in PCPhs vs. 3.4 ± 2.2 in PCNs, *P* = 0.251). The age of smoking initiation was not significantly different between PCPhs and PCNs (*P* = 0.086).

**Table 1:** Smoking habits of primary care physicians and nurses

Smoking habits	PCPhs (%, means ± SD)	PCNs (%, means ± SD	*P*
Smoking status			
Current	34.4 %	30.7 %	
Former	14.0 %	10.1 %	<0.001
Non-smoker	51.5 %	59.3 %	
Duration of smoking (year)	14.6 ± 7.2	12.1 ± 6.2	<0.001
Age of smoking initiation (year)	21.7 ± 5.1	20.6 ± 4.3	0.086
Age of smoking cessation (year)	34.2 ± 6.5	29.3 ± 6.2	<0.001
Amount of cigarette a day (unit)	19.2 ± 6.6	14.8 ± 8.9	<0.001
FNDT	3.7 ± 2.7	3.4 ± 2.2	0.251

*FNDT* Fagerstrom nicotine dependence test, *SD* standard deviation, *PCPhs* Primary care physicians; *PCNs* Primary care nurses. P represented statistical value of variables between primary care providers. For statistical analysis, chi-square test was used to compare categorical variables, and student-*t* test was used to compare continues variables. *P* < 0.05 vas accepted as statistical significant

Table 2 indicates intention of PCPs to give up smoking. When comparing participants’ contemplation of current smokers about giving up smoking, no significant difference was observed (*P* = 0.103).

**Table 2:** “Intention to give up smoking” of primary care physicians and nurses

Intention to give up smoking	PCPhs (%)	PCNs (%)	*P*
Ready to quit smoking right now	22.1	18.2	0.103
Ready to quit smoking within next 6 months	44.7	37.2	
Not ready to quit smoking within next 6 months	40.7	37.1	

*P* represented statistical value of variables between primary care providers. For statistical analysis, Fisher’s exact test was used to compare categorical variables, *P* < 0.05 vas accepted as statistical significant

When knowledge level of smoking effects on health was compared among PCPs, it is important to highlight that knowledge of this issue is very high amongst both groups. The knowledge level about effect of smoking harms was high in both groups of PCPs and not statistically different (*P* = 0.098). Conversely, knowledge level on neonatal effect of passive smoking (90.4 vs. 86.1 %; *P* < 0.001), cardiac effect of passive smoking (97.1 vs. 95.6 %; *P* = 0.043), effect of paternal smoking on children (98.7 vs. 96.9 %; *P* = 0.002) and effect of maternal smoking on offspring (94.6 vs. 88.9 %; *P* < 0.001) were significantly higher among PCPhs, compared to PCNs (Table 3).

**Table 3:** Knowledge level about smoking effects of primary care physicians and nurses

Health effects of smoking	PCPhs (%)	PCNs (%)	*P*
Neonatal effect of passive smoking	90.4	86.1	<0.001
Harmful health effects of smoking	98.9	98.3	0.098
Cardiac effect of passive smoking	97.1	95.6	0.043
Effect of paternal smoking on exposed children	98.7	96.9	0.002
Effect of maternal smoking during pregnancy on offspring	94.6	88.9	<0.001

*P* represented statistical value of variables between primary care providers. For statistical analysis, Fisher’s exact test was used to compare categorical variables, *P* < 0.05 vas accepted as statistical significant

A comparison of attitudes toward anti-smoking interventions in PCPhs and PCNs, shown in Table 4, revealed significant differences for some items. Role modeling (96.2 vs. 90.1 %), asking about smoking habits (87.3 vs. 80.1 %), advising quitting smoking (89.2 vs. 80.7 %), training in smoking cessation practices (86.6 vs. 78.3 %), banning of sponsorships supported by the tobacco industry (88.7 vs. 86.5 %), the usefulness of pharmacotherapy in smoking cessation (59.5 vs. 46.6 %), and the value of advice in increasing the chance of quitting (86.6vs. 79.8 %) were significantly higher for PCPhs (*P* < 0.001, <0.001, <0.001, <0.001, 0.008, <0.001, and <0.001, respectively).

**Table 4:** Altitudes towards smoking of primary care physicians and nurses

Altitudes towards smoking	PCPhs (%)	PCNs (%)	*P*
Role model of health provider for patients and public	96.2	90.1	<0.001
Setting prototype by not smoking	91.8	89.9	0.332
Routine asking about patients’ smoking habits by PCPs	87.3	80.1	<0.001
Routine advise patients to quit smoking by PCPs	89.2	80.7	<0.001
Getting a specific training on cessation	86.6	78.3	<0.001
Speaking to community groups about smoking	70.1	69.8	0.212
Prohibition of smoking in closed public area	93.5	93.7	0.749
Health warning on cigarette package	89.5	89.3	0.935
Banning sponsorship supported by tobacco industry	88.7	86.5	0.008
Extension of ban on the tobacco product advertising	90.9	90.1	0.737
Sharp increase the price of tobacco product	69.7	70.8	0.504
Advice patients to avoid smoking around their children	97.7	97.5	0.292
Pharmacotherapy is useful for smoking cessation	59.5	46.6	<0.001
Less likely to advise people to stop smoking, if HCPs smoke	55.3	52.5	0.386
Increase in chance of quitting smoking advised by HCPs	86.6	79.8	<0.001

*P* represented statistical value of variables between primary care providers. For statistical analysis, Fisher’s exact test was used to compare categorical variables, *P* < 0.05 vas accepted as statistical significant

**Fig. 1:**
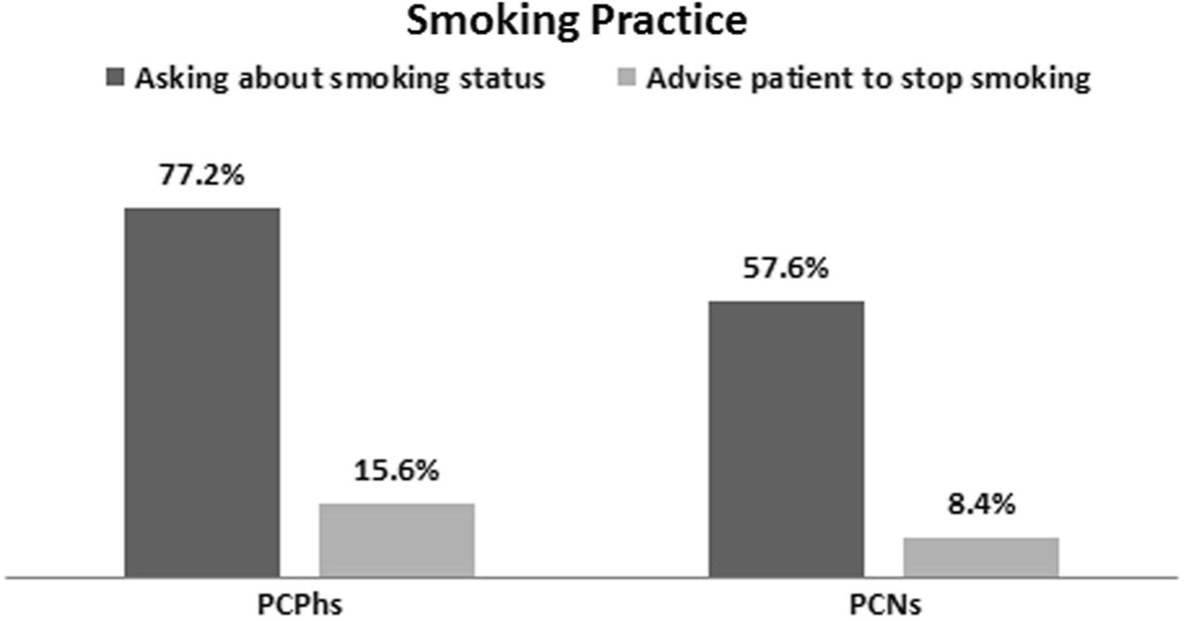
Demonstrated that “Ask” and “Advise” steps of 5A Smoking Cessation Practice implemented by primary care physicians (PCPhs) and nurses (PCNs)

The comparisons of smoking cessation practice regarding “Ask” and “Advice” steps between PCPhs and PCNs were given as overall in Fig. 1 and as detail in Table 5. Figure 1 indicates that 77.2 % of PCPhs and 58.4 % of PCNs regularly or sometimes practiced an “Ask” step (*P* < 0.001). One-third of PCPhs (33.8 %) stated that they always asked their patients about their smoking status, whereas only 27.6 % of PCNs regularly did so (*P* < 0.001). Small numbers of PCPhs (15.6 %) and PCNs (8.4 %) had advised their patients to stop smoking (*P* < 0.001). Of PCPhs, 13.1 % advised all smoker patients to quit, and 2.5 % of PCPhs advised those with relevant medical conditions to do so, whereas 6.3 % of PCNs advised all smoker patients and 2.1 % of PCNs advised those with relevant medical conditions to quit (*P* < 0.001). Table 5 shows In Table 5, the vast majority of PCPhs stated that they were felt competent regarding advising about smoking interventions, but only a minority of PCNs considered themselves competent (75.1 vs. 17.3 %; *P* < 0.001).

**Table 5:** “Ask” and “Advise” steps of smoking cessation practice implemented by primary care physicians and nurses

Smoking cessation practice steps	PCPhs (%)	PCNs (%)	*P*
Asking about smoking status			<0.001
Regularly always asking	33.8	27.6	
Sometimes asking	43.4	30.8	
Never	22.8	41.6	
Advise patient to stop smoking			<0.001
Advise to all smokers	13.1	6.3	
Advise to smokers with relevant medical conditions	2.5	2.1	

*P* represented statistical value of variables between primary care providers. For statistical analysis, chi-square test was used to compare categorical variables, *P* < 0.05 vas accepted as statistical significant

**Fig. 2:**
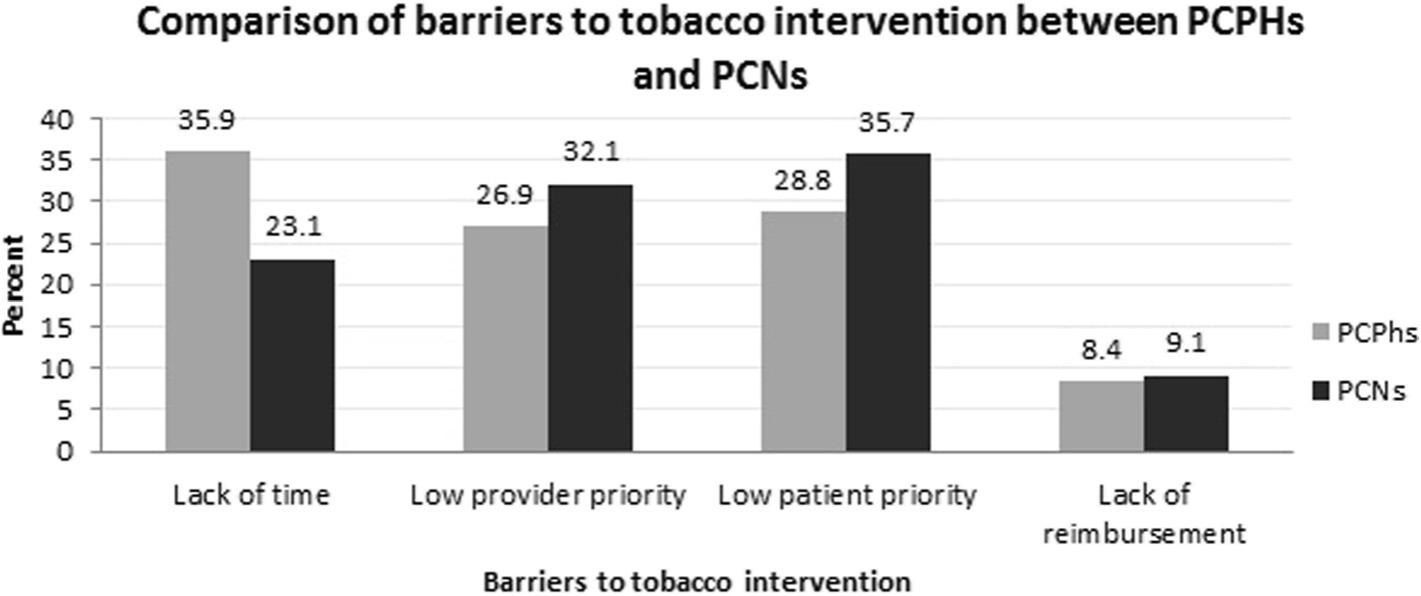
Demonstrated that barriers to tobacco intervention stated by primary care physicians and nurses: Lack of time (35.9 %) was predominantly stated by primary care physicians (PCPhs) and low patient priority (35.7 %) was predominantly stated by primary care nurses (PCNs) (*P* < 0.001)

Regarding barriers to tobacco intervention, lack of time, low patient priority, low provider priority, and lack of reimbursement were cited by 35.9, 28.7, 26.9, and 8.4 % of PCPhs and by 23.1, 35.7, 32.1, and 9.0 % of PCNs, respectively. Lack of time was the item most commonly cited by PCPhs, whereas low patient priority was most commonly cited by PCNs (*P* < 0.001; Fig. 2).

## Discussion

This study’s findings are based on data from healthcare providers working in primary care settings in the several cities in Turkey; thus, they are likely representative of healthcare providers nationwide. The findings provide a comprehensive and comparative look at tobacco use, tobacco attitudes, and knowledge and practice among PCPhs and PCNs in primary care settings in Turkey.

Based on our findings, smoking prevalence among PCPs (34 % for PCPhs and 30 % for PCNs) was higher in our country than in some other countries. In the USA, a study conducted in 2010 found that smoking prevalence was less than 6 % among PCPhs and less than 13 % for nurses [13]. Stamatopoulou et al. [14] reported that smoking prevalence among nurses was 32 % in Greece. A study conducted in Bosnia and Herzegovina in 2002 revealed that approximately 45 % of surveyed physicians and nurses smoked [15]. A study in 2000 of Italian general practitioners determined that 28.3 % smoked [16]. The smoking prevalence in the present study was markedly higher than that in the general Turkish population according to a 2010 study (TURDEP II), which revealed that the smoking prevalence was about 31 % in men and 10 % in women [17]. We emphasize that there was comparable difference in ratio of current smoking between the two PCP groups. The majority of the PCPhs in our study was male, but almost all of the PCNs were female staffs. That’s why the statistical significant was observed between two groups.

The ages of smoking initiation and cessation and the duration and amount of smoking in our participants were similar to those of the general population. Health professionals had shorter smoking durations, earlier smoking cessation, and lower smoking amounts than did PCPhs in this study. We suppose that the number of female primary care providers was greater among healthcare professionals than in the general population, and female physicians are less likely to smoke. This would explain the shorter duration, earlier cessation, and lower amount of smoking among healthcare professionals. The BREATHE study revealed that the average smoking amount, in terms of pack-years, was lower among females than males [18]. Bernat et al. [19] stated that 25 % of young adults initiated smoking between the ages of 18 and 21 years, in contrast to our results. Consistent with our results, the age of initiating smoking in the general population was reported in a systemic review to be 18–24 years [20]. In our study, the differences in age of smoking cessation and amount of cigarette a day between two PCP groups were observed. We can suggest that differences might be due to heavier work load and more stress factors among PCPhs than PCNs.

An important point was PCPs’ “intention to give up smoking” in this study and similarities in this item. Measures of the intention to stop smoking vary among countries, and the range of responses and ethnicity [21]. Tsoh et al. [22] reported that 36 % of their subjects intended to quit soon. In our study, a minority of PCPhs and PCNs stated that they were ready to quit smoking right now. Smit et al. [23] suggested that desire and intention were independent predictors of quit attempts, whereas duty was not a predictor. Apart from the duty of PCPs to quit, measures of intention to stop smoking along with attempts among PCPs may help them change their behavior.

Health professionals are expected to be role models for their patients, and that includes, in general, their behavior in health-related matters such as diet and exercise, particularly tobacco use [24]. Health professionals have the opportunity to model healthy behavior for their patients [25, 26]. We observed that items related to attitudes toward being a role model and setting a good example by not smoking were significantly different between PCPhs and PCNs in this study, and at least 90 % of PCPhs and PCNs had positive attitudes toward serving as a role model for their patients and the public and setting a good example by not smoking. Consistent with our these findings, another study reported that about 59.1 % of PCP staffs had positive attitudes toward smoking cessation, whereas 17.3 % had negative attitudes. We found high positive attitudes about smoking cessation and tobacco control. We suggest that this provides a good opportunity for ministerial officers to engage healthcare professionals in smoking cessation interventions by providing tailored training in such interventions. We observed that there was a profound difference in attitudes between PCPhs and PCNs are more interesting. Actually, we did not expect the significant differences in attitudes because both groups PCP groups are responsible for tobacco control in primary health care. We considered that discrepancy in faculty curriculum on smoking cessation practice and smoking interventions for physicians and nurses before and after graduation might be effective on significant differences in attitudes.

In the present study, over half of the healthcare professionals asked about the smoking status of their patients, but only about one-third of PCPhs and PCNs regularly practiced an asking step during their daily clinical activities. A study from the Mediterranean region reported that 60 and 36 % of PCPhs regularly practiced asking and advising steps, respectively, in their practices [27]. Smith et al. [28] found that that almost all nurses had asked and advised, if only seldom, but less than half did so frequently; they also reported no significant difference between rural and urban nurses.

A recent study reported five main barriers to smoking interventions by PCPhs: limited perceived role for PCPhs, lack of time during consultations, past experience and presence of disincentives, patients’ inability to afford medications, and lack of training in smoking cessation skills [29]. In a previous study, the majority of PCPhs felt that smoking cessation support was too time consuming [30]. In the present study, we found that a lack of time on the part of PCPhs and low patient and provider priority on the part of PCNs were the most commonly reported barriers. In contrast to our findings, Block et al. [31] in the USA found that low patient priority was the major issue for both PCPhs (36.5 %) and PCNs (56.8 %). In Turkey, about 3654 are affiliated with family health units, and about 65 patients per day are examined by each PCPhs [32]. The lack of time identified by PCPhs in the study may simply be due to this high workload. Although preventative medicine, such as tobacco control, is included among the responsibilities of PCNs, the low provider priority may be due to inadequate training and low competence among PCPs [33, 34]. The low patient priority may be due to resistance to quitting and a lack of awareness on the part of patients.

### Study limitations

The study had some limitations. The limitations of self-selection and the self-report nature of the survey represent potential sources of bias and may have resulted in underestimation of the true smoking prevalence rate and misrepresentation of attitudes toward smoking and smoking cessation practice. Self-report questionnaires are always open to respondent bias, especially on a sensitive topic such as smoking behavior. Participation in this study was voluntary, and current smokers may have avoided completing the study survey or participating in the study at all. The results of this study are not fully representative of PCNs and PCPhs in primary care settings across Turkey, and should not be generalized to healthcare professionals more broadly. Some socio-demographic features of healthcare providers, including marital status and economic levels, could have considerable effects on behavior and knowledge levels of healthcare providers regarding tobacco control; we did not include marital and economic information of participants in the analyses. Finally, passive smoking has been considered another problem for human health, and we did not investigate whether PCPs asked their patients about passive smoking exposure.

## Conclusions

The rate of current smoking among primary care providers in Turkey is higher than that in many countries. There were differences and similarities regarding smoking habits between primary care physicians and nurses. Knowledge levels and attitudes toward smoking and tobacco control were high among all primary care providers, but higher among physicians than among nurses. Barriers to smoking intervention most frequently stated by physicians and nurses were lack of time and low patient priority, respectively. Smoking interventions by healthcare providers were quite low. The majority of physicians felt competent, but nurses did not, regarding smoking intervention measures.
